# Female Gender Scheme is Disturbed by Polycystic Ovary Syndrome: A Qualitative Study From Iran

**DOI:** 10.5812/ircmj.12423

**Published:** 2014-02-07

**Authors:** Fatemeh Nasiri Amiri, Fahimeh Ramezani Tehrani, Masoumeh Simbar, Reza Ali Mohammadpour Thamtan, Niloofar Shiva

**Affiliations:** 1 Department of Midwifery and Reproductive Health, Faculty of Nursing and Midwifery, Shahid Beheshti University of Medical Sciences, Tehran, IR Iran; 2 Reproductive Endocrinology Center, Research Institute for Endocrine Sciences, Shahid Beheshti University of Medical Sciences, Tehran, IR Iran; 3 Department of Biostatistics, School of Health Sciences, Mazandaran University of Medical Sciences, Sari, IR Iran; 4 Reproductive Endocrinology Center, Research Institute for Endocrine Sciences, Shahid Beheshti University of Medical Science, Tehran, IR Iran

**Keywords:** Polycystic Ovary Syndrome, Qualitative Research, Hirsutism, Gender Identity, Femininity

## Abstract

**Background:**

Polycystic ovary syndrome (PCOS) is the most common endocrinopathy affecting up to one in every five women of reproductive age. The majority of researches on PCOS focus on its biomedical aspects, often overlooking and neglecting women’s own perceptions and experiences.

**Objectives:**

This study aimed to explore women’s perception and experiences that influence their personal gender role.

**Patients and Methods:**

This research is a qualitative study by conventional content analysis. Semi-structured interviews were conducted with 23 reproductive aged women with PCOS, recruited from the reproductive endocrinology research center. , in-depth interviews were continued to reach data saturation. The study was carried out at the reproductive endocrinology research center of Shahid Beheshti University in Tehran All the interviews were recorded and transcribed, and qualitative content analysis of the data was conducted manually.

**Results:**

Four themes were identified. Content analysis of the interviews revealed these women mainly perceived themselves with lack of physical attractiveness, loss of womanhood, interruption of sexual role and disruption of fertility potential, feelings were related to symptoms e.g. ‘excess’ hair; absent or disrupted menstrual cycle, obesity and infertility commonly experienced by women with PCOS.

**Conclusions:**

Women with PCOS are challenged in their perceptions of themselves as “feminine” because of their hairy appearance, irregular menses and lack of fertility and this influences their gender roles. Medical practitioners must understand how PCOS precisely affects women’s roles and initiate management aimed at reconstructing their “womanhood”, along with their medical treatment.

## 1. Background

Polycystic ovary syndrome (PCOS) is the most common gynecological endocrinopathy (1, 2), with a prevalence ranging between 2.2% to 26%, depending on the definitions used for diagnosis (3-6). PCOS, a heterogeneous disorder that influences reproductive ability and metabolic functioning and related disorders (7, 8), is difficult to diagnose because of the variation in its presentation (7-9). There are no clear and contemporaneous criteria for its recognition, however women with PCOS will generally experience one or more of the following symptoms in varying degrees: Menstrual irregularities, infertility, obesity, hirsutism, male pattern hair loss and acne (7, 10, 11).

Women with PCOS were not only threatened by the clinical aspects and long term medical complications of this disease, but their “womanhood” is also completely disrupted, and the embarrassment of physical unattractiveness and shame of the possible ‘deviation’ from proper femininity”, because of lack of the ability, makes them feel abnormal to bear children (12). In PCOS women, alterations in the physical and aesthetic standards (hirsutism, obesity, acne, and alopecia) and an imbalance of sexual hormones negatively impact their sexual role, often disrupting their sexual functions, by affecting their self-esteem and female identity (13, 14). Studies demonstrate that a diagnosis of PCOS has a negative impact on psychosocial and emotional well-being of these women (15). Despite the heavy burden and impact of PCOS on the well-being of women, there are limited studies addressing the experiences of women with PCOS that demonstrate perceptions of these women regarding their identity and gender roles.

## 2. Objectives

This study aimed to investigate the perception of the Iranian PCOS women regarding their gender scheme.

## 3. Patients and Methods

### 3.1. Participants and Data Collection

A total of 23 in-depth interviews were conducted, between June and March 2011, to explore and document perceptions of women with PCOS, about their underlying disease and its influences on their gender roles. Diagnosis of PCOS syndrome by Rotterdam criteria is based on having at least two of the following three features: 1) Oligo or an ovulation, 2) Clinical and/or biochemical signs of hyperandrogenism, and 3) Polycystic ovaries ( 16 ). The inclusion criteria included in this study: they were aged 18 to 40, met the Rotterdam criteria for diagnosis of PCOS and willing to participate and share their experiences. The exclusion criteria included the coexisting illness, inability to read and speak Persian, and having conditions similar to PCOS at presentation, such as congenital adrenal hyperplasia. Because if they have participated in our study patients by ultrasound and physical examination and were diagnosed by gynecologists and endocrinologists. Study participants were women with PCOS, aged 18-40 years, who had been referred to the Reproductive Endocrinology Research Center and after being informed about the objectives and process of the study were willing to participate and share their experiences. They were a heterogeneous group with various socioeconomic backgrounds and clinical phenotypes (Table 1) and the exclusion/inclusion criteria were being able to speak Persian and not have history of any other chronic medical disorders or psychopathology. Interviews were conducted by the main researcher and lasted between 45 to 90 min (on average 75 min), in a private room using a semi-structured guide questionnaire consisting of open-ended questions, enabling respondents to fully explain their perceptions and experiences.

**Table 1.** Characteristics of the Study Subjects

	No. (%)
**Age groups, y**	
18-24	8 (34.8)
25-34	13 (56.5)
35-40	2 (8.7)
**Marital status**	
Married	15 (65.2)
Single	8 (34.8)
**Educational level**	
Primary	2 (8.7)
Secondary/diploma	15 (65.2)
University	6 (26.1)
**Occupational status**	
Housewives	10 (43.5)
Employees	4 (17.4)
Self-employed	4 (17.4)
University student	4 (17.4)
Farmer	1 (4.3)
**Onset of PCOS**	
Onset of the puberty	21 (91.3)
Post pubertal/ marriage	2 (8.7)
**Main concerns**	
Infertility	8 (34.8)
Hirsutism	3 (13.05)
Acne	1 (4.3)
Menstrual irregularities	4 (17.4)
Obesity	2 (8.7)
Recurrent abortion	3 (13.05)
Hair loss	2 (8.7)

During the interview notes were written regarding the nonverbal signals; the records were the transcribed and analyzed consecutively. To begin, each participant was asked to describe her individual perception on PCOS and its influences on her daily life and her role as a “women”. All in-depth interviews were conducted, taped, transcribed and analyzed in Farsi. To establish credibility, a qualified bilingual individual, competent in the professional terminology of the women’s qualitative research, validated the quality and conceptual equivalence of the translation. Purposive sampling was used and followed with theoretical sampling according to the codes and categories as they emerged. Interviewing was stopped when data saturation occurred (17, 18); data saturation occurred when no more codes identified through the last three of interviews and when the emergent categories were coherent.

### 3.2. Data Analysis

Data collection and analysis were performed simultaneously according to the content analysis methodology. Meaning units, primary codes, sub categories, categories, themes and definition were applied to the data. During open coding, each transcript was reviewed by at least two authors and the data was reduced to codes Differences in coding were resolved via discussions; codes that were found to be conceptually similar in nature or related in meaning were grouped into subcategories. In open primary coding the intent was to clarify how the emergent subcategories were related to categories. Analytical tools included asking questions and making comparisons, and were utilized to identify the properties of each concept. 

### 3.3. The Rigor

To evaluate the rigor and enhance the trustworthiness of data, we drew on Lincoln and Guba’s (2000) criteria to evaluate qualitative data; which included credibility, transferability, dependability, and conformability. Credibility, which refers to the confidence one can have in the findings, can be established by some strategies in qualitative research (19). In this study the accuracy of data were established by in-depth prolonged engagement with participants, selection of participants with various socioeconomic backgrounds and different clinical phenotypes, member checking to confirm fitness of results by some of the participants. Member checking, an important step in guarding against researcher bias, allowed the participants the opportunity to either agree or disagree with the conclusions drawn from the interviews. Lincoln and Guba consider this the most important procedure for establishing credibility in a study. In this study; three randomly selected participants were given a full transcript of their coded interviews with a summary of the emergent themes to determine whether the codes and themes matched their point of view. The participants provided feedback and all confirmed that they were in agreement with the concepts and themes that were developed by the research team. Results were also checked with some of the women with PCOS, who did not participate in the research and they confirmed the fitness of the results as well. In addition to the members of the research team, two experts in qualitative researches, who did not participate in the research conducted a second review and confirmed the fitness of the results as well. To increase data portability, the researcher documented all of the steps followed in the research to facilitate repeating the study protocol in future research Ethics. The ethics committee of the Shahid Beheshti University of Medical Sciences approved the study. Participants provided written informed consent before the beginning of interviews, signing a consent form before audio taping.

## 4. Results

The findings of this study began via providing demographic information of the participants and continued through providing an overall impression of participants' subjective experiences and themes extracted from the interviews. Demographic information of the study participants is presented in Table 1. Based on data analysis, the major themes and sub themes of women’s gender role derived from the experiences of PCOS subjects are shown in Table 2. 

**Table 2.** Major Themes and Sub-themes

Themes	Sub-themes
**Lack of the self-perceived physical attractiveness**	Obesity
	hirsutism
	hair loss
	acne
**Loss of womanhood**	Male pattern body hair
	Bearded woman
	Masculinity
	Breast atrophy
	Not having the “normal women’s appearance”
	Dressing in male
	Not having a normal women’s social life
	Male pattern hair loss
**Disruption of sexual function**	Painful intercourse
	Loss of sexual attractiveness
	Feeling of sexual dissatisfaction
	Lack of sexual pleasure
	Lack of sexual excitement
	Lack of libido
	Sense of shame for sexual coldness
	Feeling “unfeminine” in sexual relationship
**Disruption of the fertility function**	Fear of Infertility
	Meaningless intercourse(inability to reproduce)
	Divorce for being infertile
	Feeling ashamed and guilty for being unable to bear children to continue husband’s generation

### 4.1. Lack of the Self-perceived Physical Attractiveness

The study findings showed that due to physical effects of the syndrome, especially obesity, hirsutism, hair loss, and acne, most of the women affected had poor unsatisfactory self-body images, besides considering themselves unattractive and feeling ashamed and embarrassed. In this regard, 29 years old married woman with a diploma, stated with discomfort *'I'm not happy with my appearance as I cannot wear the clothes I like, because of my obesity; I feel embarrassed when I want to go to a party or a wedding'.*

A single female student, 25 years old nervously and sadly stated *‘I was became so nervous whenever I saw the hair which made me wonder why so much hair was on my head; it made me really nervous, even when I wanted to go to the gym for swimming (as I like swimming very much), I did not go because of my hairy appearance; you cannot constantly remove the hair; well, it is difficult’.*

In addition, a 24 year old housewife mentioned the impact of acne on herself: *'Whenever I wear a dress with part of my body exposed, others look at my rashes; they zoom in on the rashes; or when you go to a gathering, everyone looks at your face rash and asks, “Why do you have all those rashes on your face?” and I don’t want to explain …'. She then continued 'I could tolerate if it was only the rash; my skin color darkens too; the rash scars remain on my face and is full of spots’.*

On the effects of this syndrome on self-perceived physical attractiveness, a 23 year old female student said *'Beauty is very important for a woman; my hair loss is really bothering me. It is too severe; my hair also reduced my beauty. I want to go to the gym but I've lost a lot of hair. I want to be in society to communicate with others; you feel very bad; only you can understand better yourself. Of course, I'm not ungrateful to God; he has blessed us with so much, but this problem exists too'.*

### 4.2. Loss of Womanhood

The effects of PCOS on sense of loss of womanhood are another subjective experience of women with polycystic ovary syndrome. In this regard, a housewife, 23 year old and diploma, stated *'My body has a coarse hair male like pattern, which my husband frankly comments on. It really affected me and this makes me very uncomfortable, at least in front of my husband'.*

Another 27 year old single female employee said *'It's bad for a woman to have a beard like the men; it's really bad; well, this is really embarrassing to be hairy and have a beard'.*

 A 27 years old married woman with diploma, stated *'I feel more masculine than feminine; I think it's because of the hormones in my body; my masculine feelings are stronger than my feminine ones'.*

A 23 year old housewife also said *'after marriage, my husband really blamed me for my masculinity; he always said “your masculine manners are more; we argued over this issue and he always blamed me for my masculine manners”; I feel I’m not a proper woman'.*

A 34 years old single female employee stated *'I feel that my breasts are small compared with other parts of the body. More often I wear males’ clothing. I like to wear blouses and trousers more. I have all men’s wear; I don’t even have a skirt; I always wear a suit. I love to be a man. I always say to God, 'I wish you didn’t give me these two (pointing to her breasts) and had given me another thing.’ I would have really loved being a boy'.*

A single 23 years old female student stated nervously *'I went to the dermatologist for hair loss. He told me your hair loss is abnormal and the cause of your hair loss is due to increased androgen. I feel the front of my hair is falling similar to male pattern baldness. This hurt me so much'.*

### 4.3. Disruption of Sexual Function

The majority of women with polycystic ovary syndrome were not satisfied with their sexual function and some had a feeling of embarrassment towards their husband due to their sexual dysfunction and coldness. In this regard, a woman 24 year old mentioned *'I’ve recently had problem with my husband regarding (sexual performance), I think all of my desire is gone. I do not feel sexual desire. I just want to finish it as soon as possible because I am not interested in physical contact with my husband and I have problems with my husband mostly because of this'.*

A housewife, 27 years old and diploma, also said* 'I think the disease has really affected my sexual performance. I do not enjoy having sex; if I didn’t have that much hair, I would enjoy my sexual life. It's really a bad feeling to be hairy; it grows very fast and annoys you wherever you want to go'.*

A 22 years old housewife with a diploma said *'I am not drawn to sexual pleasure. A lot of times I don’t like to have intercourse'.*

A 24 years old housewife with a diploma said *'I feel almost too cold for sex but my husband is so hot for sex, exactly the opposite of me. Nothing is sexually exciting for me. I am so sad to be annoying my husband and I feel ashamed because of my cold sexual attitude'.*

However a few of the PCOS women were satisfied with their sexual performance and believed that this syndrome has not had any impact on their sexual attractiveness, e.g. a 28 year old married female employee, said *'I feel that this syndrome has not affected my sexual function, my libido is better and so is my sexual desire. I am not a cold woman'.*

### 4.4. Disruption of the Fertility Function

Another feeling experienced by these women is the fear of infertility. A single woman, 32 years old and diploma, stated *'I fell I’m not being like “normal women”. There is a disruption in my body that I have not regular menstruation. I’m afraid to have problems for breeding children after getting married'.*

Some women participants of this study were already infertile due to polycystic ovary syndrome and they came to the research center because they were worried about remaining childless in the future and wished to conceive.

In this relation, a 23 year old housewife with a diploma concerned about this syndrome, said *'The fertility problem is more important to me; than the excess hair growth or obesity, I can solve the excessive hair and obesity, but not fertility problem, I feel that if I can't bear a child; I will lose all sense of being a woman'.*

A 24 year old said *'I feel that intercourse is really a farce. I feel no desire to have it, because I am not pregnant'.*

A 40 year old housewife stated *'My husband fights with me a lot and has constantly called me” infertile". It is now 10 years that he doesn’t like to come home'.*

A 29 years old housewife said *'I’d like to have a child because my husband is very keen to have his son carry on his name'.*

## 5. Discussion

This research indicates that PCOS women are perceptions of themselves as “ordinary women” are challenged. Lack of the self-perceived physical attractiveness, loss of womanhood, disrupted sexual function and infertility function were four major themes extracted. The majority of women participants mentioned that the syndrome's symptoms, had negative impacts on their self-perceived physical attractiveness and body image and this led to the feeling of embarrassment and lack of self-confidence. Hopwood et al. (2001), explained, for the first time, that body image is an important goal in evaluating the quality of life in the treatment of cancers which cause major changes in patient’s appearances due to surgery, delayed effects of radiotherapy, or side effects of systemic medications (20).

 In an overview study by Deeks et al. (2010), it was reported that having the polycystic ovary syndrome is a disappointing experience for women and is associated with challenges to their feminine identity and body image due to obesity, acne and excess hair, as well infertility and its long-term health related concerns and a compromised quality of life that adversely impacts mood and psychological well-being (21). In a qualitative study, Ekback et al. (2009) evaluated the experiences of women with hirsutism and reported that most women participants did not have a good self-body image and considered themselves ugly and unattractive which led to low self-esteem and social interaction among them. They eventually concluded that hirsutism can cause severe emotional distress, negatively impacting their quality of life and limiting their personal and social life (22). Polycystic ovary syndrome is one of the common causes of hirsutism in 60% of cases (23). Likewise, Trent et al. (2002) found that most common symptoms of PCOS such as menstrual irregularities, obesity, male pattern of body hair growth, acne and other skin problems play a major role in impaired body image, self-confidence and decreased quality of life (24), findings similar to those of the present study.

The loss of feminine characteristics and the appearance of masculine features was one of the gender role’s effect that repeatedly mentioned by the study participants. In a qualitative study by Kitzinger and Willmott (2002) investigating the experiences of British women with PCOS, three themes were extracted which included feelings of being" freakish, abnormal and non-proper ". Smooth hairless bodies and faces, regular menstruation and the capacity to bear children were considered as signs of femininity by the affected women, as a result of which symptoms, these women expressed feeling different from other women and felt less feminine. They concluded that polycystic ovary syndrome creates an obviously undesirable condition in sense of femininity so much so that as the syndrome is called "the thief of womanhood" (12).

Likewise, in a study by Tressa et al. (2011), one of the major concerns of women with PCOS has been reported to be an assault on their feminine characteristics due to hirsutism, alopecia (androgenetic baldness) and weight gain (25). In this study, women considered themselves physically less than other women and felt disappointed, angry, and embarrassed because of the syndrome induced changes. In a qualitative study by Barbara Snyder (2006), women with PCOS described the feelings of being different from others and hirsutism negatively impacted their sense of femininity (26). Boomsma and et al. (2006) has reported that the syndrome leads to the deepening of patients' voice due to increased androgens, as well as to shrinking breasts and increased muscle mass in the body, findinds in agreement with experiences of Iranian women (27).

Our results revealed that the majority of women with PCOS experience some problems in their sexual function mainly owing to the effects of this syndrome on their physical appearance. Elsenbruch et al. (2003) also reported that changes in the physical and aesthetic aspects of women’s bodies (hirsutism, obesity, acne, and alopecia) and imbalance in sex hormones contributes to reduced quality of life and sexual function in these patients (13). Two studies have found that pain during sexual intercourse is increased in women with PCOS compared to controls (28, 29). The incidence of painful intercourse seems to be negatively influenced by BMI (28). In addition, insufficient lubrication was significantly higher in PCOS women (30), a finding which seems to explain the higher incidence of painful sexual intercourse. The incidence of sexual thoughts and fantasies (part of subjective arousal) seems to be negatively correlated to BMI (28); however, orgasm frequency was not found to differ between PCOS women and controls (29). It is widely recognized that women with PCOS report decreased sexual satisfaction than healthy controls (13, 29, 31-33). Sexual satisfaction seems to be influenced by both endocrine and psycho-social factors, e.g, both BMI and hirsutism seem to negatively influence both sexual satisfaction and sexual attractiveness (28).

PCOS women also thought that their partners found them less sexually attractive (31). Sexual self-worth seems to be lower in PCOS women (31, 34). Lower body-image has been found to be associated with sexual avoidance (35). There is evidence that sex typed behavior and sexual orientations are related to hormonal levels as one study measured sex-typed behavior by internet survey as well as self-reported PCOS diagnosis (36). The results indicated that PCOS women reported significantly less typical feminine behavior as a child (e.g., experimenting with make-up). In addition, PCOS women reported to have lower rates of dating boys and being part of a sports team. Women with PCOS seem to have lower self-esteem and lower body image compared to the general population (37). Overall decreased sexual satisfaction has been reported by PCOS women and it is negatively correlated to BMI and hirsutism (13, 29, 31-33).

In our study, women reported fear of infertility in the future and present. In agreement with our study, Trent et al. (2003) in a cross-sectional study reported that girls with PCOS were 3.4 times more likely than healthy girls to be “worried about their ability to become pregnant in the future” compared to healthy adolescents (68% vs. 41%), with controlling for age (32). Infertility was found to have negative effects on marital relations due to one of the spouses wanting to divorce or separation (38).Our data shows that infertility affects the relationships of the couples because the infertile spouse feels guilty and considers separating or getting divorced for not being able to give a child to his partner. Most of the participant of present study emphasized the importance of having a child as they really felt the absence of a child in their relationship. Marriage, the fundamental function of which is to sustain generations, is one of the basic behaviors of humanity. Views of the women participants of this study pertaining to marriage and having children correlate with the data available.

Regarding limitations of this study are the nature of the sample, which consisted of volunteers recruited from reproductive endocrinology research center. It is also possible that the experiences reported by volunteer participants might have been more positive than those who chose not to be interviewed. Limitations of this study were overcome by the use of purposive sampling as the sample was more heterogeneous demographically, especially in educational attainment and PCOS manifestations. The small sample is not necessarily representative of all Iranian women with PCOS. To explore how different cultural contexts shape experiences of PCOS to view and their symptoms, further research with a more diverse sample using the findings of cultural studies of femininity, beauty, and body image is recommended. Even if previous research suggests that PCOS causes emotional distress and affects quality of life in a negative way, the complexity of the problem previously has not been fully understood or described. In summary, our study shows that PCOS thoroughly affects the women’s ideas about their bodies. The women were constantly preoccupied with their hairiness, obesity and infertility which greatly affected their ideas about themselves. Polycystic ovary syndrome is a stressful heterogeneous disease, requiring therefore, social support interventions; however little is known about the content of such interventions or patient’s experiences of using them. More qualitative research is needed to identify social support needs in PCOS, and the extent to which these may be met by various forms of intervention.
